# Association Between Sugar-Sweetened Beverage Consumption and Psychological Distress: Analysis of the Nutrition and Health Survey in Taiwan

**DOI:** 10.3390/nu18183037

**Published:** 2026-09-17

**Authors:** Wei-Ching Huang, Jia-Yau Doong

**Affiliations:** 1 Department of Nutrition and Health Sciences, Fooyin University, Kaohsiung 831301, Taiwan; AA646@fy.edu.tw; 2 Department of Nutrition, College of Medicine, I-Shou University, Kaohsiung 82445, Taiwan

**Keywords:** sugar-sweetened beverages (SSBs), dietary pattern, mental health, psychological distress, Nutrition and Health Survey in Taiwan (NAHSIT)

## Abstract

Objective: This study aimed to assess the association between sugar-sweetened beverage (SSB) consumption and psychological distress. Design: This study used a cross-sectional design. Setting: The database from the 2013–2016 Nutrition and Health Survey in Taiwan (NAHSIT) was used for this study. Participants: There are 3787 Taiwanese adults aged 19–64 in the NAHSIT database. Measurements: The simplified food frequency questionnaire was used to assess the frequency of SSB intake, and a 24 h dietary recall was used for nutrient calculations. Subsequently, a logistic regression model was used to examine the association between the frequency of SSB consumption and psychological distress. Result: The risk of psychological distress with SSBs consumed 1–6, 7–13, and ≥14 times per week was (adjusted odds ratio (aOR) = 1.75, 95% CI = 1.50–2.04), (aOR = 1.56, 95% CI = 1.32–1.84), and (aOR = 1.99, 95% CI = 1.64–2.42), respectively. Participants who consumed SSBs ≥14 times per week had a significantly lower intake of whole grains, milk, vegetables, fruits, and nuts and seeds, while their consumption frequency of eggs, dairy products, soybean products, pickled vegetables, meat products, tea, coffee, desserts and snacks, and fried foods was significantly higher. Conclusions: SSB consumption was significantly positively associated with psychological distress. Future interventional studies could further explore the potential directionality of this association.

## 1. Introduction

Mental health is an important aspect of public health and is closely related to physical health [1]. Studies have found a positive association between mental health and dietary behavior [2], particularly with the consumption of sugar-sweetened beverages (SSBs), which is thought to have a certain impact on mental health [3,4,5]. In Korean adults, consuming at least one SSB per day is linked to a higher risk of both depression and suicidal ideation [6]. Daily consumption of SSBs is significantly associated with poor mental health, including experiencing more days of poor mental health per month, while drinking 100% fruit juice has no such association [3].

The study found that during the COVID-19 pandemic, 36% of U.S. adults increased their consumption of unhealthy snacks and desserts at times, and 16% did so frequently, while 22% of adults increased their consumption of SSBs at times, and 10% did so frequently [7]. However, consuming excessive sugar may produce addictive behaviors, leading to an imbalance in dopamine and serotonin metabolism, which in turn affects appetite and emotional disorders [8]. Regular SSB intake may also lead to neurobehavioral adaptations that reinforce consumption patterns, further exacerbating mental health issues [4].

Long-term intake of sweet foods and beverages, including SSBs, is associated with an increased risk of common mental disorders and recurrent depression, particularly in men [5]. The combined consumption of SSBs and fast foods has a more detrimental impact on mental health than consuming either alone. This association includes increased stress, depressive symptoms, and suicidal ideation [9]. A meta-analysis demonstrated that a higher frequency of SSB consumption is associated with a significant increase in the risk of depression, following a non-linear dose–response pattern. Compared to individuals who did not consume SSBs, those who consumed the equivalent of two cups of SSBs per day exhibited a 5% higher risk of depression, while consuming the equivalent of three cans of SSBs was associated with a 25% increased risk of depression [10].

Previous studies have explored the association between SSBs and mental health in Western countries, but there are few relevant studies in Asian countries [11]. Moreover, studies often have limitations due to varying dietary patterns across different cultures and populations. These dietary patterns can significantly influence the association between SSB consumption and psychological distress, potentially leading to inconsistent or non-generalizable results. Despite the established physical health risks of SSBs, their influence on mental health, particularly in Asian populations, has not been thoroughly investigated. Therefore, this study used Taiwan’s nationally representative database to explore the association between SSB intake and psychological distress.

## 2. Methods

### 2.1. Participant Data Sources

The data source is the nationally representative Nutrition and Health Survey in Taiwan (NAHSIT) 2013–2016 database provided by the Health and Welfare Data Science Center (Application No.: H113253). The detailed study design and sampling methods are published elsewhere [12]. In total, 3850 participants, ranging in age from 19 to 64, participated in face-to-face household interviews. Of these participants, 3493 also underwent physical examinations. Among the NAHSIT, 3787 participants completed the simplified food frequency questionnaire (SFFQ) and the Brief Symptom Rating Scale (BSRS-5). This study was approved by Antai Medical Care Cooperation Antai Tian-Sheng Memorial Hospital Institutional Review Board (24-089-C).

### 2.2. Dietary Information

Dietary information was collected using the SFFQ, which included questions about how often 79 food items were consumed in the past month. Its reliability and validity have been published in a previous study [13]. In this study, food items were divided into 15 food groups, and the food frequency intake was computed weekly. Nutrient intake was obtained from 24 h dietary recall data in the database, and nutrient density was calculated per 1000 kcal.

### 2.3. Sugar-Sweetened Beverages (SSBs)

Data on the frequency of SSB intake were obtained from the following SFFQ question in the database: “How often do you consume any sugary drinks (including lactic acid beverages, carbonated beverages, sports drinks or functional beverages)?”. The frequency of intake was subsequently calculated weekly for this study. This study divided the frequency of SSBs into no intake, 1–6 times, 7–13 times, and ≥14 times/week.

### 2.4. Psychological Distress

The BSRS-5, developed by Dr. Ming Been Lee, is widely used in a non-psychiatric facility setting to identify minor psychological disorders associated with depression and anxiety [14]. It was also included in the NAHSIT 2013–2016 as part of the household interview. This tool consists of 5 questions that ask participants to reflect on the last week and indicate the level of distress they experienced, including (1) trouble falling asleep, (2) feeling tense or high-strung, (3) feeling easily annoyed or irritated, (4) feeling depressed or in a low mood, and (5) feeling inferior to others, with each question rated on a scale of 0–4 (0, not at all; 1, a little bit; 2, moderately; 3, quite a bit; and 4, extremely). The score range is as follows: 0–5 points indicate good physical and mental condition; 6–9 points indicate mild psychological distress; 10–14 points indicate moderate psychological distress; and ≥15 points indicate severe psychological distress [15]. The internal consistency (Cronbach’s alpha) coefficient of BSRS-5 ranges from 0.77 to 0.90. The test–retest reliability coefficient is 0.82 [16]. To align with clinically relevant general distress, this study binarized the scores and defined psychological distress as ≥6 points.

### 2.5. Statistical Analysis

Data analysis was performed using SAS statistical software (version 9.4, SAS Institute, Cary, NC, USA). Continuous variables are expressed as mean ± standard error, while categorical variables are expressed as percentages. In general, linear regression was used to compare differences in the frequency of SSB intake in the distribution of continuous variables such as baseline demographic variables, frequency of intake of food groups, and nutrient intake. The Chi-square test was used to compare differences in the distribution of categorical variables.

A multiple logistic regression model was used to explore the association between SSBs and psychological distress. All regression analyses incorporated complex sampling weights, strata, and clusters using survey-specific procedures to ensure valid population-level estimates. Except for BMI, all missing covariate values were less than 4%. Therefore, the categorical variables were imputed as the mode, while the total daily caloric intake was imputed as the mean. The missing value of BMI was 46%, and to avoid the bias of large imputation, five databases were generated using multiple imputations (under the Missing at Random assumption, using sex and age as predictors) [17], which showed no substantial changes in the estimated odds ratios. Variance Inflation Factors (VIFs) indicated no significant multicollinearity (all VIFs < 2.5). All tests were two-tailed, and *p* < 0.05 indicated a significant difference.

## 3. Results

The results indicate that higher SSB consumption is more frequent among men and individuals with middle personal income, smoking habits, and higher psychological distress scores. Conversely, SSB consumption greater than 14 times per week is notably lower among older individuals who have higher perceived health status and use dietary supplements. Furthermore, the prevalence of psychological distress in this study was found to be 12.5% (Table 1).

When analyzed by sex, among men with a university education or higher, 14.2% consumed SSBs ≥ 14 times per week, which was higher than the other education groups, while no significant difference was observed in alcohol consumption. In contrast, higher education levels in women were associated with a lower frequency of SSB intake. Additionally, smoking and alcohol consumption were linked to a higher frequency of SSB intake among women (*p* < 0.001) (Tables S1 and S2).

Table 2 presents data on SSB intake and dietary information. The results indicate that individuals who consumed SSBs ≥ 14 times per week had significantly lower protein and dietary fiber intake but higher energy and fat intake. Furthermore, their consumption frequency of whole grains, milk, vegetables, fruits, nuts, and seeds was significantly lower. Conversely, they had a significantly higher intake frequency of eggs, soybeans and products, processed dairy products, pickled vegetables, meat and their products, tea, coffee, sweets, and fried foods.

Table 3 explores the association between SSB intake frequency and psychological distress using multiple logistic regression. The results of Model 1 (adjusted for age, sex, and strata) indicate that participants who consumed SSBs 1–6 times, 7–13 times, and ≥14 times per week had a 1.75-fold (95% CI: 1.50–2.04), 1.56-fold (95% CI: 1.32–1.84), and 1.99-fold (95% CI: 1.64–2.42) higher risk of psychological distress, respectively, compared to those who had no intake of SSBs. These risks increased significantly with the frequency of SSB intake (*p* for trend < 0.001). The results of Model 4 (adjusted all possible potential covariates) indicate that participants who consumed SSBs 1–6, 7–13, and ≥14 times per week had a 1.77-fold (95% CI: 1.50–2.08), 1.53-fold (95% CI: 1.28–1.82), and 1.93-fold (95% CI: 1.57–2.37) higher risk of psychological distress, respectively, compared to those who had no intake of SSBs. A sensitivity analysis was used to compare the data before the imputation of model 1, which found no significant differences in this study and showed no substantial changes in the estimated odds ratios. These individual item analyses utilized binary cutoffs (score ≥ 2) to indicate moderate to severe distress. In addition, a higher frequency of SSB consumption was significantly associated with an increased risk of individual psychological distress symptoms, including feeling tense, easily annoyed, depressed, and inferior to others (all *p* for trend < 0.01) (Table S3).

## 4. Discussion

The results of this study indicate that among Taiwanese adults aged 19–64, those who consume SSBs ≥ 14 times per week have approximately a 2.0-fold increased risk of psychological distress compared to no intake. Furthermore, individuals who consume SSBs more frequently tend to have higher energy and fat intake. In contrast, their protein and dietary fiber intake is significantly lower compared to those who do not consume SSBs. Additionally, the frequency of whole grain, milk, vegetable, fruit, nut, and seed consumption is significantly lower in individuals who consume SSBs.

Longitudinal studies show that SSB consumption is significantly associated with an increased risk of depressive symptoms with a dose–response pattern [18,19]. Among Mexican adults aged 18–40 years, self-reported executive function problems may influence their intake of SSBs by causing emotional distress such as anxiety and depression [20]. A cross-sectional study was conducted with 3667 adults in China; the results show that high consumption of soft drinks was significantly related to a higher prevalence of depressive symptoms among adults [21]. This evidence suggests a significant association between high consumption of SSBs and an increased risk of psychological distress, including depression and common mental disorders. Therefore, reducing the intake of SSBs may be associated with better long-term psychological health, though causality cannot be inferred from this cross-sectional study.

Even low to moderate consumption of SSBs was linked to increased fasting glucose levels, high-sensitivity hs-CRP [22], reduced LDL particle size, and a more atherogenic LDL subclass distribution, which promotes inflammation [23]. Inflammation biomarkers show significant alterations in multiple psychiatric disorders, including depression, post-traumatic stress disorder, and sleeping disorders [24]. Moreover, in prediabetes, individuals with high consumption of sugar-sweetened beverages have increased inflammation, such as C-reactive protein (CRP) levels [25]. Therefore, increased intake of SSBs may promote biomarkers of inflammation and worsen mental health.

Long-term sugar consumption can alter serotonin receptor responsiveness, potentially affecting mood and stress adaptation [26]. Serotonin and dopamine systems are crucial in the pathophysiology and treatment of major mental disorders, including depression and schizophrenia [27]. Serotonin inhibits dopaminergic function, and this interaction is significant in the therapeutic effects of certain antipsychotics [28]. Dysfunction in these signaling mechanisms can contribute to depressive symptoms.

High consumption of SSBs is not only related to mental health problems [3] but also increases the risk of developing metabolic syndrome, type 2 diabetes, and lower bone mineral density [29]. The observed associations were dependent on the amount consumed, with higher intake of SSBs associated with increased risks of stroke, depression, cancer, and all-cause mortality for each additional 250 mL per day [30]. In addition, habitual SSB consumption may be part of an overall unhealthy lifestyle that includes poor diet, sedentary behavior, and other factors that contribute to metabolic dysregulation and mental health problems [9,30].

Our results show that there is no association between the frequency of SSB intake and BMI. Studies have found a positive association between consumption of SSBs and BMI [31,32]. However, a systematic review of the results showed that the association between SSB consumption and BMI was inconsistent, whereas the intake of milk was negatively associated with BMI [33].

In this study, the consumption of whole grains, milk, vegetables, fruits, nuts, and seeds was significantly lower among individuals who frequently consumed SSBs. Conversely, these individuals exhibited a significantly higher intake frequency of eggs, soybeans and their products, processed dairy products, pickled vegetables, and meat and meat products, as well as tea, coffee, sweets, and fried foods. Those who purchased SSBs daily or weekly were less likely to consume two or more servings of fruits and vegetables per day [34]. A systematic review of observational studies shows that fruit and vegetable consumption had a positive influence on mental health [35]. A prospective cohort study found that dietary patterns characterized by a high intake of chocolate, sweets, and cream and a low intake of fruits and vegetables were associated with symptoms of depression and anxiety. Another dietary pattern was associated with a higher intake of SSBs and added sugars and a lower intake of cream/cheese, but it was not significantly associated with depression and anxiety [36].

This study has several strengths and potential limitations. The NAHSIT is a nationally representative database; therefore, it can be used to estimate the general population in Taiwan. However, this study is cross-sectional and thus cannot explain cause and effect. Specifically, there is a potential for reverse causality, as psychological distress may precede and drive SSB intake; individuals experiencing psychological distress may consume high-sugar foods as a coping mechanism for emotional regulation (comfort eating). Furthermore, the SFFQ did not ask about participants’ consumption of SSBs qualitatively, so it is not possible to know each individual’s usual intake volume or the exact amount of free sugar per serving. In addition, participants might have severe psychiatric symptoms but are unwilling to report them, which may lead to incorrect group assignments. We also lacked data on confounders such as physical activity, previous psychiatric diagnoses, and total caffeine intake (which is bundled within the SSB category). The multiple statistical comparisons were not corrected for multiple testing, increasing the risk of false positives. We adjusted for BMI as a potential confounder rather than a mediator, given that baseline adiposity may influence both diet and mental health, though it could partially act on the causal pathway. Additionally, as demonstrated by our results, high SSB intake often co-occurs with lower overall diet quality (such as reduced intake of whole grains and fruits and higher intake of processed foods), making it difficult to fully isolate its independent effect from broader dietary habits.

## 5. Conclusions

Consumption of SSBs is associated with an increased prevalence of psychological distress and tends to be associated with suboptimal dietary patterns. These findings highlight the association between SSB intake and mental health, though longitudinal studies are required to establish causality. Therefore, future research needs to further clarify the causal relationship between SSB intake and mental health and explore the mechanism.

## Figures and Tables

**Table 1:** Baseline characteristics by sugar-sweetened beverage intake among individuals in the NAHSIT 2013–2016 (N = 3787).

	Sugar-Sweetened Beverage Intake Frequency				
	No Intake	1–6	7–13	≥14	*p* for Trend
N, %	520 (13.7)	2003 (52.9)	922 (24.4)	342 (9.03)	
Sex, %					<0.001
Men	12.6	48.3	26.8	12.3	
Women	14.9	57.5	21.9	5.8	
Age (years)	52.0 ± 0.48	43.9 ± 0.30	38.5 ± 0.42	35.6 ± 0.67	<0.001
19–30	4.10	46.6	33.2	16.1	
31–50	9.01	53.8	27.9	9.35	
51–64	25.3	56.3	14.6	3.84	
Education, %					<0.001
Illiterate	28.6	53.6	14.3	3.57	
Primary and below	20.3	52.9	21.0	5.83	
Secondary and high	13.3	52.9	24.5	9.27	
University and above	7.95	52.6	28.1	11.4	
Smoking, %	12.6	47.1	25.8	14.4	<0.001
Drink alcohol, %	16.1	53.3	22.7	7.9	<0.001
Personal income, %					<0.001
<20,000	16.0	53.1	23.3	7.66	
20,001–49,999	9.60	52.3	26.9	11.3	
≥50,000	17.7	54.5	22.1	5.73	
Perceived health status, %					<0.001
Good	17.0	53.3	22.2	7.53	
Fair	12.1	54.2	24.7	9.06	
Poor	13.4	50.1	25.9	10.6	
Sleep duration (h)	7.45 ± 0.06	7.53 ± 0.07	7.58 ± 0.05	7.68 ± 0.08	0.013
Supplement use, %	12.9	50.7	26.4	10.1	<0.001
BMI (kg/m^2^)	24.2 ± 0.21	24.2 ± 0.13	24.6 ± 0.22	24.4 ± 0.39	0.227
Waist circumference (cm)	84.0 ± 0.54	83.4 ± 0.31	84.0 ± 0.49	84.6 ± 0.81	0.398
Percentage of body fat, %	32.5 ± 0.50	32.2 ± 0.27	31.9 ± 0.45	30.4 ± 0.79	0.038
BSRS-5 score	1.65 ± 0.10	2.34 ± 0.07	2.44 ± 0.10	2.53 ± 0.17	<0.001

Abbreviation: BSRS: Brief Symptom Rating Scale BSRS-5. Categorical variables are presented as percent, and continuous variables are presented as mean ± SEM. A general linear regression and the Chi-square test were used for continuous and categorical variables.

**Table 2:** Comparisons of food intake frequency and sugar-sweetened beverage intake in the NAHSIT 2013–2016.

	Sugar-Sweetened Beverage Intake Frequency				
	No Intake	1–6	7–13	≥14	*p* for Trend ^‡^
Energy (Kcal)	1858 ± 32.5	1927 ± 16.8	2016 ± 25.7	2189 ± 46.2	<0.001
Nutrient density/per 1000 kcal					
Protein (g)	42.7 ± 0.54	42.1 ± 0.25	40.5 ± 0.38	39.8 ± 0.66	<0.001
Fat (g)	32.7 ± 0.48	34.1 ± 0.23	34.4 ± 0.34	34.7 ± 0.54	0.004
Carbohydrate (g)	132 ± 1.38	130 ± 0.65	132 ± 0.94	129 ± 1.43	0.314
Dietary fiber (g)	11.3 ± 0.27	9.05 ± 0.11	7.29 ± 0.15	5.97 ± 0.20	<0.001
Food intake frequency, weekly					
Whole grain	3.27 ± 0.23	1.98 ± 0.08	1.26 ± 0.10	0.74 ± 0.11	<0.001
Egg	3.43 ± 0.15	3.91 ± 0.07	4.79 ± 0.11	5.22 ± 0.21	<0.001
Milk and powder	1.84 ± 0.13	1.76 ± 0.06	1.45 ± 0.08	1.54 ± 0.15	0.004
Processed dairy products	0.76 ± 0.08	0.88 ± 0.04	0.99 ± 0.06	1.24 ± 0.13	<0.001
Soybean and products	4.55 ± 0.18	4.72 ± 0.08	5.01 ± 0.17	5.11 ± 0.26	0.013
Vegetables	26.8 ± 0.75	25.7 ± 0.33	24.7 ± 0.47	25.4 ± 0.79	0.044
Pickled vegetables	0.55 ± 0.05	0.73 ± 0.03	0.83 ± 0.06	1.12 ± 0.11	<0.001
Fruits	8.44 ± 0.29	7.02 ± 0.13	5.67 ± 0.16	4.41 ± 0.28	<0.001
Nuts and seeds	1.93 ± 0.13	1.60 ± 0.06	1.09 ± 0.07	1.08 ± 0.11	<0.001
Fish and seafood	7.48 ± 0.26	7.27 ± 0.13	7.61 ± 0.20	7.96 ± 0.35	0.092
Meat and products	7.12 ± 0.26	8.70 ± 0.14	11.2 ± 0.24	14.0 ± 0.51	<0.001
Tea	5.05 ± 0.47	4.09 ± 0.14	4.67 ± 0.16	7.83 ± 0.40	<0.001
Coffee	2.23 ± 0.18	2.03 ± 0.07	2.96 ± 0.11	4.09 ± 0.33	<0.001
Desserts and snacks	2.48 ± 0.15	3.98 ± 0.13	4.63 ± 0.17	5.19 ± 0.33	<0.001
Fried food	0.30 ± 0.03	0.72 ± 0.02	1.28 ± 0.05	1.71 ± 0.11	<0.001

All variables are presented as mean ± SEM. Total daily energy intake excluding unreasonable data (men < 800 kcal or >4200 kcal; women < 500 kcal or >3500 kcal). Nutrient intakes are standardized per 1000 kcal. ^‡^ *p* for trend was estimated using a general linear regression model.

**Table 3:** The association between sugar-sweetened beverage intake frequency and risk of psychological distress, based on the 2013–2016 NAHSIT (N = 3787).

	Sugar-Sweetened Beverage Intake Frequency				
Adjust OR (95% CI)	No	1–6	7–13	≥14	*p* for Trend
Psychological distress/normal	35/485	266/1737	118/804	53/289	
Crude	1.00	2.12 (1.82–2.46)	2.03 (1.73–2.39)	2.54 (2.11–3.06)	<0.001
Model 1	1.00	1.75 (1.50–2.04)	1.56 (1.32–1.84)	1.99 (1.64–2.42)	<0.001
Model 2	1.00	1.78 (1.52–2.08)	1.54 (1.30–1.83)	1.90 (1.56–2.31)	<0.001
Model 3	1.00	1.79 (1.53–2.09)	1.55 (1.31–1.84)	1.94 (1.59–2.36)	<0.001
Model 4	1.00	1.77 (1.50–2.08)	1.53 (1.28–1.82)	1.93 (1.57–2.37)	<0.001

The odds ratio was estimated for binary logistic regression models. Model 1: adjusted for age, sex, and sampling strata. Model 2: adjusted additionally for education, smoking, drinking alcohol, personal income, perceived health status, and supplement use. Model 3: Model 2 plus energy. Model 4: Model 3 plus BMI.

## Data Availability

The datasets presented in this article are not readily available because of strict ethical and legal restrictions placed on Taiwan’s national health data to protect participant privacy. Requests to access the datasets should be directed to the Health and Welfare Data Science Center, Ministry of Health and Welfare, Taiwan.

## Supplementary Materials

The following supporting information can be downloaded at https://www.mdpi.com/article/10.3390/nu18183037/s1: Table S1. Baseline characteristics by sugar-sweetened beverage intake for adult men in the NAHSIT 2013–2016 (N = 1895); Table S2. Baseline characteristics by sugar-sweetened beverage intake for adult women in the NAHSIT 2013–2016 (N = 1892); Table S3. The association between sugar-sweetened beverage intake frequency and risk of psychological distress based on the 2013–2016 NAHSIT.
