# Supplementary material for: Association Between Sugar-Sweetened Beverage Consumption and Psychological Distress: Analysis of the Nutrition and Health Survey in Taiwan
Source: Nutrients. 2026 Sep 17;18(18):3037. doi: 10.3390/nu18183037 (PMC13610773; doi:10.3390/nu18183037)
Supplement: Supplementary file 1 [file nutrients-18-03037-s001.zip › nutrients-4527244-supplementary.pdf]

Table S1. Baseline characteristics by sugar-sweetened beverage intake in men adults among NAHSIT 2013-2016 (N=1895).

	Sugar-sweetened beverage intake frequency				
	No intake	1-6	7-13	≥14	P for trend
N, %	239 (12.6)	915 (48.3)	508 (26.8)	233 (12.3)	
Age (years)	52.0±0.66	45.0±0.44	38.7±0.57	34.7±0.78	<0.001
19-30	3.35	37.2	37.2	22.2	<0.001
31-50	8.41	48.4	29.5	13.7	
51-64	23.4	55.8	16.8	4.05	
Education, %					<0.001
Illiterate	0.00	100	0.00	0.00	
Primary and below	17.5	50.7	24.0	7.79	
Secondary and high	13.0	47.7	26.9	12.5	
University and above	6.88	50.0	28.9	14.2	
Smoking, %	13.4	47.3	24.6	14.6	<0.001
Drink alcohol, %	13.9	45.5	28.5	12.1	0.364
Personal income, %					<0.001
<20000	15.4	47.7	25.1	11.8	
20001-49999	8.57	46.6	29.4	15.4	
≥50000	16.5	51.6	25.2	6.77	
Perceived health status, %					0.013
Good	15.4	48.2	26.1	10.3	
Fair	10.3	50.7	27.1	11.9	
Poor	13.5	43.8	27.0	15.6	
Sleep duration (hour)	7.39±0.10	7.47±0.05	7.53±0.06	7.69±0.10	0.014
Supplement use, %	12.3	52.9	24.6	10.3	0.014
BMI (kg/m^2)	25.0±0.31	24.8±0.18	25.1±0.28	24.8±0.45	0.815
Waist circumference (cm)	88.3±0.79	87.5±0.43	87.3±0.64	86.6±0.93	0.198
Percentage of body fat, %	27.0±0.63	26.2±0.35	27.3±0.50	27.2±0.89	0.326
BSRS-5 score	1.42±0.15	2.02±0.09	2.10±0.13	2.17±0.19	0.007

Abbreviations: BMI, body mass index; BSRS-5, Brief Symptom Rating Scale.

Categorical variables are presented as percent, and continuous variables are presented as mean ± SEM. General linear regression and Chi-square test or Fisher's exact test were used for continuous and categorical variables.

Table S2. Baseline characteristics by sugar-sweetened beverage intake in women adults among NAHSIT 2013-2016 (N=1892).

	Sugar-sweetened beverage intake frequency				
	No intake	1-6	7-13	≥14	P for trend
N, %	281 (14.9)	1088 (57.5)	414 (21.9)	109 (5.76)	
Age (years)	51.9±0.67	43.1±0.40	38.3±0.61	37.5±1.25	<0.001
19-30	4.85	56.1	29.1	9.92	<0.001
31-50	9.60	59.1	26.2	5.08	
51-64	27.3	56.8	12.3	3.63	
Education, %					0.007
Illiterate	32.0	48.0	16.0	4.00	
Primary and below	21.8	54.2	19.3	4.73	
Secondary and high	13.7	58.4	22.0	5.90	
University and above	9.70	56.7	26.9	6.72	
Smoking, %	8.25	45.9	32.5	13.4	<0.001
Drink alcohol, %	17.3	57.5	19.5	5.64	0.002
Personal income, %					<0.001
<20000	16.4	56.1	22.2	5.34	
20001-49999	10.8	58.6	24.0	6.62	
≥50000	20.8	62.4	13.9	2.89	
Perceived health status, %					0.013
Good	19.0	59.4	17.4	4.13	
Fair	13.9	57.6	22.3	6.24	
Poor	13.4	56.4	24.9	6.37	
Sleep duration (hour)	7.50±0.08	7.58±0.04	7.63±0.07	7.67±0.13	0.181
Supplement use, %	16.9	58.0	19.4	5.67	0.018
BMI (kg/m^2)	23.5±0.27	23.7±0.18	24.0±0.34	23.5±0.71	0.558
Waist circumference (cm)	80.7±0.65	80.2±0.40	80.1±0.70	80.5±1.43	0.705
Percentage of body fat, %	37.2±0.52	36.7±0.26	37.0±0.55	37.1±0.93	<0.001
BSRS-5 score	1.85±0.15	2.61±0.09	2.87±0.16	3.30±0.35	<0.001

Categorical variables are presented as percent, and continuous variables are presented as mean ± SEM.
General linear regression and Chi-square test were used for continuous and categorical variables.
Psychiatric morbidity scores were assessed using the Brief Symptom Rating Scale (BSRS-5).

Table S3. The association between sugar-sweetened beverages intake frequency and risk of psychological distress on 2013-2016 NAHSIT.

	Sugar-sweetened beverage intake frequency				
	No	1-6	7-13	≥14	P trend
Trouble falling asleep/normal	30/487	128/1874	56/866	25/317	
Model 1	1.00	1.10 (0.92-1.30)	1.01 (0.83-1.23)	1.26 (1.00-1.60)	0.122
Model 2	1.00	1.17 (0.98-1.41)	1.11 (0.90-1.37)	1.27 (0.99-1.64)	0.214
Feeling tense/normal	5/515	45/1958	18/904	8/333	
Model 1	1.00	2.10 (1.43-3.09)	1.71 (1.13-2.61)	2.18 (1.35-3.51)	<0.001
Model 2	1.00	2.27 (1.51-3.41)	1.85 (1.19-2.89)	2.35 (1.42-3.91)	<0.001
Feeling easily annoyed/normal	8/511	65/1936	46/876	24/316	
Model 1	1.00	1.51 (1.11-2.06)	2.08 (1.51-2.87)	2.87 (2.03-4.07)	<0.001
Model 2	1.00	1.54 (1.12-2.14)	1.98 (1.41-2.78)	2.57 (1.77-3.71)	<0.001
Feeling depressed/normal	8/511	43/1959	28/894	12/329	
Model 1	1.00	1.15 (0.84-1.58)	1.47 (1.05-2.06)	1.70 (1.15-2.52)	0.006
Model 2	1.00	1.11 (0.79-1.56)	1.43 (1.00-2.06)	1.66 (1.09-2.51)	0.010
Feeling inferior to others/normal	9/510	51/1951	32/890	13/327	
Model 1	1.00	1.31 (0.97-1.76)	1.67 (1.21-2.30)	1.75 (1.21-2.54)	0.003
Model 2	1.00	1.39 (1.01-1.91)	1.72 (1.22-2.43)	1.74 (1.17-2.60)	0.007

The odd ratio was estimated for binary logistic regression models.

Model 1: adjusted for age, sex., and sampling strata.

Model 2: adjusted additionally for education, smoking, drinking alcohol, personal income, perceived health status, and supplement use, energy, and BMI.
